# Pulse Oximetry Screening for Critical Congenital Heart Defects: Effectiveness and Implementation across Clinical Settings

**DOI:** 10.26502/fccm.92920468

**Published:** 2025-08-08

**Authors:** Gauri Gurumurthy, Devendra K. Agrawal

**Affiliations:** 1 Department of Translational Research, College of Osteopathic Medicine of the Pacific; 2 Western University of Health Sciences, Pomona, California 91766 USA

**Keywords:** Atrial septal defect, Congenital heart defect, Clinical settings, Critical congenital heart defect, Efficacy, Implementation, Patent ductus arteriosus, Pulse oximetry screening, Tetralogy of Fallot

## Abstract

Congenital heart defects are the most prevalent congenital anomalies, occurring in approximately 1% of live births and accounting for nearly one-third of all major congenital disorders worldwide. Within this spectrum, critical congenital heart defects (CCHDs) represent the most severe forms, often necessitating surgical or catheter-based intervention within the first year of life. Delayed or missed diagnosis of CCHDs remains a major cause of preventable neonatal morbidity and mortality, underscoring the importance of timely detection. Pulse oximetry screening has emerged as a noninvasive, inexpensive, and highly specific method to identify hypoxemia, particularly in duct-dependent lesions that may otherwise escape early clinical recognition. Since its universal adoption in the United States in 2011, pulse oximetry screening has consistently demonstrated value as a complementary tool to prenatal ultrasound and physical examination, improving detection rates, reducing emergency hospitalizations, and lowering infant mortality. Despite its proven clinical impact, important challenges remain. Screening sensitivity is limited, especially for conditions such as coarctation of the aorta that may not produce early hypoxemia. Variability in protocols, including timing of screening, pre- versus post-ductal measurement, and thresholds for repeat testing, contributes to inconsistent performance across hospitals. Moreover, recent evidence of racial bias in pulse oximetry accuracy, along with persistent disparities in early detection across demographic groups, highlights the need for inclusive device validation and standardized protocols. In low- and middle-income countries, pilot studies from Rwanda, Nigeria, and India demonstrate feasibility and dual benefits for detecting both cardiac and non-cardiac hypoxemic conditions, but widespread implementation is hindered by resource constraints, inadequate referral pathways, and limited access to confirmatory echocardiography. Emerging strategies, including integration with electronic medical records, computerized clinical decision support systems, and mobile health technologies, hold promise for standardizing workflows, improving compliance, and extending reach to resource-limited settings. This article presents a synthesisis of current evidence on the effectiveness, limitations, and global implementation of pulse oximetry screening for the detection of congenital heart defect detection, highlighting both its established role in neonatal care and the urgent need for innovations to address disparities, optimize protocols, and ensure equitable access to screening worldwide.

## Introduction

Congenital heart defects (CHDs) are the most common type of birth defect, affecting approximately 1% of all live births. Within this spectrum, critical congenital heart defects refer to the most severe forms that require surgical or catheter-based intervention within the first year of life [1]. These include conditions such as hypoplastic left heart syndrome, transposition of the great arteries, pulmonary atresia, and tetralogy of Fallot [2–5]. Early detection of CHDs is crucial, as delayed diagnosis can lead to life-threatening cardiovascular collapse shortly after birth [6]. Advances in genomic technologies have shed light on the potential role of genetic variation, particularly de novo variants (DNVs), in the etiology of CHD. DNVs account for an estimated 8% of sporadic CHD cases, a figure that increases significantly in the presence of extracardiac anomalies or neurodevelopmental delays. Despite these advances, many genetic causes underlying sporadic CHD remain poorly understood [7]. This underscores the need not only for improved diagnostic tools but also for effective early screening programs, such as pulse oximetry, to identify newborns with CHDs before the onset of critical symptoms.

Pulse oximetry has emerged as an indispensable noninvasive screening tool for the early detection of hypoxemia and critical congenital conditions in pediatric populations. Among its most impactful uses is in the early identification of critical congenital heart defects (CCHDs). Subclinical hypoxemia in newborns with duct-dependent lesions may not present with overt symptoms, and pulse oximetry has proven effective in identifying these neonates before clinical deterioration occurs. As a result, universal pulse oximetry screening has been recommended by the American Academy of Pediatrics (AAP) and the Centers for Disease Control and Prevention (CDC) as part of routine newborn care [8].

Beyond CCHD, pulse oximetry plays a vital role in the early detection of acute respiratory illnesses, such as bronchiolitis, pneumonia, and asthma exacerbations. Studies have shown that continuous or spot SpO_2_ measurements correlate with disease severity and can guide decisions on hospitalization and oxygen therapy initiation [9]. In neonatal and pediatric intensive care units (NICUs and PICUs), continuous SpO_2_ monitoring enables clinicians to detect early signs of respiratory decompensation, apnea of prematurity, and desaturation events in critically ill patients [10].

Additionally, pulse oximetry is increasingly utilized in screening for sleep-disordered breathing, especially in children with risk factors such as obesity, craniofacial abnormalities, or neuromuscular diseases. Overnight pulse oximetry serves as a preliminary tool to detect nocturnal hypoxemia and guide the need for polysomnography or other diagnostic evaluations [11].

While pulse oximetry screening for CCHDs and other hypoxemic conditions has demonstrated significant clinical value, a comprehensive review of its effectiveness and implementation is essential to optimize outcomes and ensure equitable application. Several factors underscore the need for such an evaluation. Variability in screening performance, including differences in sensitivity and specificity, has been reported across studies and healthcare settings. These differences often arise from inconsistent screening protocols, variations in the timing of measurements, anatomical site selection, and differences in device technology. Without standardized implementation, the clinical yield of pulse oximetry may be diminished, and false-positive or false-negative results can impact diagnostic accuracy and resource utilization [12–15].

Technological and physiological limitations can influence the accuracy of pulse oximetry readings. Artifacts caused by patient movement, low peripheral perfusion, ambient light interference, and skin pigmentation can lead to erroneous readings [16]. Recent evidence has raised concerns regarding racial bias in pulse oximetry measurements, with overestimation of oxygen saturation observed in patients with darker skin tones. This may result in missed or delayed diagnosis of hypoxemia in certain populations, highlighting the need for inclusive device validation [17–18].

Health system and resource constraints play a critical role in the feasibility of widespread screening. In low- and middle-income countries (LMICs), challenges such as insufficient equipment availability, limited staff training, lack of maintenance, and inadequate referral pathways can impede effective implementation. Addressing these barriers is essential to ensure that the benefits of pulse oximetry extend to all neonatal and pediatric populations globally [19–22]. Finally, cost-effectiveness considerations must be weighed against health system priorities. Although modeling studies have demonstrated that routine CCHD screening with pulse oximetry is cost-effective, especially when integrated into existing newborn screening programs, the initial investment in devices, training, and follow-up systems may be prohibitive in under-resourced settings [22–23].

A critical review of the literature can inform policymakers and clinicians on strategies to improve implementation efficiency while maintaining clinical effectiveness. The association between pulse oximetry screening and early detection of critical congenital heart defects reflect a crucial intersection of neonatal care, preventive cardiology, and health systems implementation. This article provides detailed examination of the current evidence on the effectiveness of pulse oximetry in identifying critical CHD, the limitations and challenges of its widespread adoption, and the potential for improved outcomes when integrated into standardized newborn screening protocols.

## Overview of Congenital Heart Defects (CCHD)

Congenital Heart Defects (CHD) refers to structural or functional abnormalities of the heart or great vessels present from birth, resulting from disruptions in cardiac morphogenesis during embryonic development. CHD is the most common type of major congenital anomaly, accounting for nearly one third of such defects worldwide. The most practical measure of its occurrence is birth prevalence, expressed as cases per 1,000 live births. A comprehensive systematic review and meta-analysis of 114 studies, covering more than 24 million live births across multiple continents, found that reported CHD birth prevalence increased substantially over the past century—from 0.6 per 1,000 live births (95% CI: 0.4–0.8) in the early 1930s to 9.1 per 1,000 (95% CI: 9.0–9.2) after 1995—stabilizing over the last 15 years at approximately 1.35 million affected newborns annually. This upward trend is largely attributed to improved diagnostic capabilities and screening programs, although true incidence may also be influenced by genetic, environmental, and socio-economic factors. Geographic variation is notable: Asia reports the highest birth prevalence (9.3 per 1,000; 95% CI: 8.9–9.7), with proportionally more pulmonary outflow obstructions, while Europe reports significantly higher prevalence than North America (8.2 vs. 6.9 per 1,000; p <0.001) [24–28].

Disparities in healthcare access, prenatal care coverage, and diagnostic infrastructure likely account for much of this variation, particularly between high- and low-income countries. Advances in cardiovascular diagnostics and pediatric cardiac surgery have transformed survival, leading to an emerging and growing adult population with Grown-Up Congenital Heart Defects (GUCH), estimated at 4 per 1,000 adults, many of whom require lifelong specialized care. These trends underscore CHD as an enduring global health challenge with substantial implications for healthcare planning, resource allocation, and screening policy [29].

Prenatal diagnosis of CHD provides critical opportunities for clinical planning and family counseling. Identification of cardiac anomalies during pregnancy enables healthcare providers to offer guidance regarding prognosis, plan optimal obstetric follow-up, and arrange delivery in tertiary care facilities equipped with specialized neonatal cardiac services, including therapeutic catheterization and cardiovascular surgery. Such coordinated care has been associated with reductions in both morbidity and mortality among affected newborns [30–31]. Despite these benefits, global data reveal that prenatal detection rates remain suboptimal. Even in high-resource countries, detection rates for CHD range between 30% and 60%. Moreover, diagnosis often occurs late in gestation; for instance, in Belgium, only 14.1% of CHD cases are diagnosed before 25 weeks, while in Argentina the mean gestational age at diagnosis is nearly 30 weeks [32–33].

Congenital heart defects represent a heterogeneous group of structural abnormalities, ranging from minor lesions that may remain asymptomatic to critical malformations that pose immediate threats to neonatal survival. Clinically, these defects are often classified into cyanotic and acyanotic lesions, a distinction based on the presence or absence of systemic desaturation, or into critical and non-critical CHDs, depending on the urgency of intervention required. This classification not only guides clinical management but also highlights the strengths and limitations of screening modalities such as pulse oximetry, which is particularly effective in detecting cyanotic and ductal-dependent lesions. A structured overview of the major categories provides a foundation for understanding the epidemiology, diagnostic challenges, and implications for early screening [35–36].

Acyanotic defects include septal defects, patent ductus arteriosis, and obstructive lesions. Septal defects are among the most common congenital heart defects, characterized by abnormal openings in the interatrial or interventricular septum that permit shunting of blood between the cardiac chambers. Ventricular septal defects (VSDs) are the most prevalent, accounting for 20–25% of all CHDs, and can vary widely in size, number, and anatomical location, including perimembranous, muscular, supracristal, atrioventricular, and the rare Gerbode type. Their natural history is diverse: while many small VSDs close spontaneously, larger defects may result in complications such as pulmonary vascular disease, infundibular stenosis, or aortic insufficiency. The clinical spectrum ranges from asymptomatic murmurs in small defects to overt congestive heart failure in larger ones, with echocardiography serving as the cornerstone for diagnosis and longitudinal assessment. Management strategies depend on the size, hemodynamic impact, and progression of the defect, with options spanning medical therapy, transcatheter closure, and surgical intervention [37–39].

Atrial septal defects (ASDs) are another common subtype of septal anomalies, defined by an abnormal communication between the left and right atria. They are typically classified into secundum, primum, sinus venosus, and the rare coronary sinus type, with secundum ASDs being the most frequent. The magnitude of the left-to-right shunt depends on defect size and atrial compliance, and while many patients remain asymptomatic during childhood, larger or unrecognized ASDs may lead to complications such as right atrial and ventricular dilation, pulmonary hypertension, arrhythmias, and paradoxical embolism later in life. Diagnosis is most often made via echocardiography, with color Doppler demonstrating interatrial flow. Management ranges from conservative observation in small defects to percutaneous device closure or surgical repair in cases with significant shunts or complications [40–42].

Patent ductus arteriosus (PDA) is a common congenital heart defect characterized by the failure of the ductus arteriosus, a fetal vascular connection between the pulmonary artery and the descending aorta, to close after birth. PDA accounts for a significant proportion of congenital heart defects, with reported birth prevalence varying across geographic regions and healthcare systems. Epidemiological studies indicate that PDA is more frequently diagnosed in preterm infants, particularly those with very low birth weight, where incidence can be as high as 30–60% [43]. In term infants, the overall prevalence of isolated PDA is lower, estimated at 1 in 2,000 live births, but it contributes meaningfully to the global burden of congenital heart defects due to its potential for morbidity if untreated. While small PDAs may remain asymptomatic, larger defects can result in pulmonary over-circulation, congestive heart failure, and increased risk of infective endocarditis. Advances in neonatal intensive care, echocardiographic diagnostics, and transcatheter closure techniques have significantly improved survival and long-term outcomes for affected infants. However, global disparities in access to timely diagnosis and intervention remain, underscoring PDA as both a clinical and public health challenge in the spectrum of congenital heart defects [44–46].

Cyanotic congenital heart defects are a subset of CHDs characterized by right-to-left shunting of blood, leading to systemic circulation of poorly oxygenated blood and resulting in clinical cyanosis. These defects are generally more severe, presenting in the neonatal period or early infancy, and require timely recognition and intervention to prevent significant morbidity and mortality. Among the most common cyanotic heart defects are transposition of the great arteries (TGA), in which the aorta and pulmonary artery are transposed, and tetralogy of Fallot (TOF), a complex defect that includes ventricular septal defect, right ventricular outflow tract obstruction, overriding aorta, and right ventricular hypertrophy. Both conditions represent critical congenital heart defects that are often identified through pulse oximetry screening and remain a major focus of neonatal cardiac care worldwide [47–48].

Transposition of the great arteries (TGA) is a critical congenital heart defect characterized by ventriculo-arterial discordance, in which the aorta arises from the right ventricle and the pulmonary artery from the left ventricle, leading to parallel rather than sequential circulation. This defect is life-threatening in neonates, as adequate oxygenation becomes dependent on inter-circulatory mixing through the ductus arteriosus, foramen ovale, or ventricular septal defect [49–51]. The prognosis of infants with TGA is heavily influenced by the timing of diagnosis: a large multicenter study comparing 250 postnatally diagnosed neonates with 68 prenatally diagnosed cases demonstrated significantly lower preoperative mortality (0% vs. 6%), reduced postoperative mortality (0% vs. 8.5%), and shorter hospital stays in the prenatal group (24±11 vs. 30±17 days) [52]. Importantly, infants diagnosed postnatally were more likely to present in poor condition, with higher rates of metabolic acidosis, multiorgan failure, and need for mechanical ventilation. Once admitted, management protocols, including prostaglandin E1 infusion, balloon atrioseptostomy, and arterial switch operation, were comparable between groups, suggesting that the survival advantage stems primarily from earlier recognition and optimized perinatal care. These findings underscore the critical role of prenatal screening for TGA, as early detection enables planned delivery in specialized centers, immediate stabilization, and timely surgical intervention, thereby markedly improving neonatal outcomes [49–51].

Tetralogy of Fallot (ToF) is the most common cyanotic congenital heart defect, with an incidence of approximately 0.326 per 1,000 live births [53]. It is classically defined by four features: a ventricular septal defect, right ventricular outflow tract obstruction, an overriding aorta, and right ventricular hypertrophy, all of which result from abnormal embryologic development of the interventricular septum. Advances in diagnosis, particularly fetal and postnatal echocardiography, have enabled early detection and characterization of the severity of pulmonary stenosis, aortic override, and associated anomalies, with transthoracic echocardiography serving as the cornerstone for pre-operative and long-term evaluation [54]. Surgical repair, first performed in the mid-20th century, remains the mainstay of treatment, typically involving closure of the ventricular septal defect and relief of right ventricular outflow obstruction, often with a transannular patch. While long-term survival after repair is excellent, patients are prone to complications such as pulmonary regurgitation, right ventricular dilation or dysfunction, arrhythmias, and need for re-interventions, particularly pulmonary valve replacement. Modern imaging techniques, including three-dimensional echocardiography, strain imaging, and cardiac MRI, are increasingly important in risk stratification, guiding the timing of interventions, and optimizing lifelong follow-up of patients with repaired ToF [55].

Critical congenital heart defects (CCHDs) are the subgroup of CHDs that typically require surgical or catheter-based intervention within the first year of life. These include transposition of the great arteries, tetralogy of Fallot, and truncus arteriosus, among others. Many CCHDs present with hypoxemia soon after birth, often before overt clinical symptoms appear. Pulse oximetry screening plays a vital role in this context, as it can detect low oxygen saturation—one of the earliest signs of these life-threatening conditions—allowing for timely diagnosis, intervention, and improved neonatal outcomes.

## Pulse Oximetry Screening: Clinical Utility in the United States

Pulse oximetry screening for critical congenital heart defects (CCHD) is performed by comparing pre-ductal and post-ductal oxygen saturation measurements, which allows for the detection of differential oxygenation caused by ductal shunting. The evidence base supporting this method highlights its strong diagnostic performance, with studies demonstrating favorable sensitivity and specificity in identifying CCHD, as well as an acceptable positive predictive value. When compared to physical examination and prenatal ultrasound screening, pulse oximetry offers an important complementary approach that improves overall detection rates. Importantly, implementation of this screening strategy has been associated with improved clinical outcomes, including earlier surgical intervention and reductions in infant mortality [56–58].

In 2011, the U.S. Secretary of Health and Human Services recommended the addition of critical congenital heart defects (CCHD) to the Recommended Uniform Screening Panel, thereby introducing pulse oximetry screening (POS) as a universal point-of-care newborn screening test. This policy decision reflected growing evidence that hypoxemia can serve as an early indicator of CCHD and that timely detection facilitates interventions associated with improved outcomes [59,60]. A consensus algorithm, subsequently endorsed by the American Academy of Pediatrics and other professional organizations, provided the framework for implementation across state newborn screening programs. However, multiple algorithms for CCHD newborn screening have been implemented across states, with the AAP-endorsed protocol being the most widely adopted, though variations such as those in New Jersey and Tennessee highlight trade-offs between sensitivity, specificity, cost, and ease of use. These differences underscore challenges in balancing early detection with minimizing false-positives and resource burden. Successful implementation also requires rigorous staff training to reduce misinterpretation, and studies suggest that simplified protocols or computer-based tools could improve accuracy. Despite near-universal adoption of CCHD screening by 2015, state-level data collection and surveillance remain fragmented due to limited funding and infrastructure, with many programs relying on voluntary or manual reporting. This lack of standardized, comprehensive data hampers the ability to evaluate screening outcomes, refine protocols, and optimize quality improvement, underscoring the need for robust national data collection systems [61–66].

The primary advantage of using pulse oximetry to screen for CHDs lies in its ability to detect mild hypoxemia, a common but often clinically occult feature of many CCHD lesions that may be missed during routine examinations. Large multicenter trials, such as the Saxony study including over 42,000 newborns, have demonstrated that pulse oximetry screening (POS) substantially reduces the diagnostic gap, lowering the proportion of infants with delayed diagnosis of CCHD to just 4.4% [59]. The method is noninvasive, inexpensive, and simple to perform, typically conducted between 24–72 hours of life by measuring pre- and post-ductal oxygen saturations. Implementation has extended beyond single-center research settings to statewide and national newborn screening programs in Europe and the United States, where it has been shown to achieve high specificity (~99.9%) with relatively few false positives, while facilitating timely initiation of life-saving interventions such as prostaglandin infusion and surgical repair. By integrating POS into routine postnatal care across primary, secondary, and tertiary facilities, healthcare systems have improved early recognition of ductal-dependent lesions, thereby reducing morbidity and mortality associated with late presentation of CCHD [67–70].

A large retrospective study of 77,148 newborns evaluated the American Academy of Pediatrics (AAP) critical congenital heart defects (CCHD) pulse oximetry screening algorithm. The current algorithm demonstrated high specificity (99.96%) but low sensitivity (14.3%), with a false-positive rate of 0.043%. While only one true-positive case of CCHD was detected, over 30% of false-positives revealed other significant non-CCHD conditions, highlighting the broader clinical utility of screening. A simulated modified algorithm (one repeat test instead of two) maintained the same sensitivity while slightly increasing the false-positive rate to 0.054%, suggesting that modest adjustments may improve detection of clinically important disease without substantially reducing accuracy [71, 72].

In addition to evaluating screening performance at the hospital level, population-based analyses have examined the impact of state-mandated POS policies on infant outcomes. A multi-state study using inpatient data from Arizona, California, Kentucky, New Jersey, New York, and Washington (2010–2014) assessed emergency hospitalizations among infants with CCHD before and after implementation of mandated POS. Among 9,147 CCHD emergency hospitalizations, there was a 22% decline in emergency admissions among non-Hispanic Caucasian infants following POS implementation, whereas the decline was significantly attenuated in non-Hispanic African American infants, who experienced 65% less reduction compared to Caucasians [73,74]. These findings suggest that while POS mandates may reduce preventable emergency hospitalizations overall, racial disparities persist, highlighting the need for further research to address inequities in early detection and access to timely care. While individual centers report low case detection rates, population-level data demonstrate significant mortality reduction with mandatory screening. Ongoing challenges, such as false-negatives, data reporting gaps, and variability in implementation, highlight the need for refinement of screening algorithms to maximize benefit while minimizing harm [74,75].

The introduction of universal pulse oximetry screening in 2011 in the US represented a landmark advancement in newborn care, enabling earlier recognition of critical congenital heart defects and improving survival outcomes. Evidence from both clinical trials and population-based studies underscores its value as a simple, cost-effective, and noninvasive adjunct to traditional screening methods. Nonetheless, important challenges remain, including variability in state-level implementation, inconsistent data collection, and disparities in detection across racial and demographic groups. While refinements to screening algorithms and reporting infrastructure are still needed, pulse oximetry has unequivocally established itself as a cornerstone of neonatal screening, reducing diagnostic delays and contributing to meaningful declines in infant morbidity and mortality associated with CCHD. These developments, however, must also be understood within a global context, where resource limitations, infrastructure barriers, and competing health priorities shape the feasibility and impact of screening programs. The following section will explore case examples from low- and middle-income countries (LMICs), such as Rwanda and India, to illustrate how pulse oximetry screening can be adapted and implemented in diverse health system settings.

## Implementation of Pulse Oximetry for screening newborns across Diverse Healthcare Settings

While pulse oximetry screening (POS) has become standard practice in many high-income countries, its adoption in low- and middle-income countries (LMICs) presents both unique opportunities and challenges. Implementation in resource-limited settings requires consideration of infrastructure, workforce capacity, and competing health system priorities. However, several pilot studies and national initiatives demonstrate the feasibility and impact of POS in diverse LMIC contexts [76,77].

In sub-Saharan Africa, early implementation studies have shown encouraging results. A prospective study conducted in district hospitals in Rwanda evaluated the use of POS among newborns in facilities with limited access to echocardiography or pediatric cardiology. The screening was found to be both feasible and acceptable to healthcare providers and families, even in rural communities where healthcare resources were sparse. Importantly, the study highlighted not only the early detection of critical congenital heart defects (CCHD), but also the identification of other neonatal conditions such as sepsis and pneumonia, conditions which are also associated with hypoxemia. This dual benefit illustrates how POS may extend beyond CCHD detection to strengthen newborn care more broadly in LMICs. Despite challenges-including inconsistent equipment availability and limited access to follow-up echocardiography-the program demonstrated proof-of-concept for POS as a practical, low-cost intervention adaptable to sub-Saharan African settings [78].

Evidence from Nigeria further illustrates both feasibility and diagnostic performance of POS. A large prospective study conducted in Lagos screened 6,120 newborns with POS within the first 24–48 hours of life. The investigators reported a sensitivity of 75%, specificity of 99.5%, positive predictive value (PPV) of 50%, and negative predictive value (NPV) of 99.9%. While the moderate PPV reflected limited availability of confirmatory testing, the very high NPV demonstrated the reliability of POS for ruling out CCHD in newborns. Feasibility was also a key finding, as screening was successfully conducted by nursing staff in busy postnatal wards after minimal training. The Nigerian study highlights the potential for task-shifting in settings where pediatric cardiologists and neonatologists are scarce, though it also emphasized the need for referral pathways to ensure that screen-positive infants receive timely echocardiography [79,80].

In China, a model-based cost-effectiveness analysis of postnatal pulse oximetry screening was conducted across three regions with differing socioeconomic status: Beijing (metropolitan region), Shandong (developed region), and Gansu (less developed region). The study simulated hypothetical annual birth cohorts and calculated the incremental costs per averted disability-adjusted life years (DALYs). Results demonstrated that adding pulse oximetry to clinical assessment was highly cost-effective in Beijing (Int$7,833/DALY), cost-effective in Shandong (Int$27,780/DALY), but not cost-effective in Gansu (Int$167,407/DALY), largely due to differences in accessibility to timely diagnosis and treatment [82]. Beyond CHD, screening also identified non-cardiac neonatal conditions such as pneumonia and sepsis, which are particularly relevant in low-resource settings. The findings suggest that implementation of pulse oximetry screening should initially target metropolitan and developed regions with greater healthcare infrastructure, while in less developed regions, broader investment in pediatric cardiac care and financial protection for families is urgently needed to make such screening programs both feasible and impactful [82].

Similarly, in India, pilot implementation projects have demonstrated that POS can be successfully integrated into high-volume maternity wards with minimal disruption to routine newborn care. Studies in both urban and rural hospitals have shown high acceptability among staff and families, with detection of not only critical CHD but also significant non-cardiac hypoxemic conditions. A large cross-sectional study from a community hospital in North India evaluated 19,009 newborns and identified 70 with major CHDs, including 26 critical cases. Pulse oximetry alone detected 55.7% of major CHDs and 84.6% of critical CHDs, while its addition to routine clinical examination significantly improved sensitivity for both major (from 35.7% to 75.7%) and critical CHDs (from 11.5% to 84.6%). Although specificity was relatively low, influenced by the lack of repeat testing, the high burden of infections, and limitations of oximeter performance, the study demonstrated the substantial added value of POS as a complementary screening tool in LMIC health systems [82].

Further evidence from Kerala, India, highlights the complementary role of prenatal diagnosis and peri-partum planning in improving outcomes for neonates with CCHD. In a prospective observational study conducted at a tertiary pediatric cardiac facility, 119 neonates with critical CHD requiring intervention were enrolled. Of these, 39 (32.8%) had received a prenatal diagnosis. The study revealed significant differences in pre-operative clinical stability between the two groups. Neonates with prenatal diagnosis had superior physiological status at admission, as measured by lower California modification of the Transport Risk Index of Physiological Stability (Ca-TRIPS) scores (median 6 vs. 8; p <0.001) and lower pre-operative assessment of cardiac and hemodynamic status (PRACHS) scores (median 1 vs. 3; p <0.001) [83]. These findings indicate that prenatal diagnosis enabled timely referral and optimized peri-partum care, which translated into earlier interventions and fewer pre-operative complications.

Mortality patterns further underscored the impact of prenatal detection. While overall mortality was 10%, preoperative mortality was disproportionately higher among infants diagnosed only after birth (10% vs. 2.6%), with most deaths attributable to suboptimal pre-operative stability that precluded surgical intervention. Although post-operative mortality remained low (2.5%), these findings demonstrate how prenatal diagnosis can significantly influence survival trajectories in LMIC settings where healthcare resources are limited and neonatal transport systems are often inadequate. Importantly, this study emphasizes the synergistic role of prenatal diagnosis with postnatal screening strategies such as POS. Together, these approaches can create a more comprehensive safety net for early CHD detection, allowing for both anticipatory care planning and timely postnatal identification of missed cases [83,84].

Despite these successes, barriers to large-scale POS adoption in LMICs remain significant. Limited access to confirmatory echocardiography and referral centers constrains the full clinical benefit of early detection, while high rates of home births preclude universal implementation. Although combining pulse oximetry with physical examination clearly improves detection of critical CHD, the relatively low specificity and potential for large numbers of false positives risk overwhelming already strained health systems with limited diagnostic and treatment capacity. Additional obstacles include inadequate neonatal transport infrastructure, a shortage of trained personnel, and poor awareness among pediatricians due to limited exposure during training. Consequently, nationwide screening is not currently feasible in many LMICs. Instead, a stepwise, region-specific approach-targeting states or districts with stronger health systems is recommended as a pathway toward incremental scale-up.

Future progress will depend on strengthening pediatric cardiac programs, developing public–private partnerships, expanding workforce training, and embedding CHD screening into broader child health initiatives, such as routine immunizations and comprehensive newborn screening programs. Adaptations tailored to local capacity such as simplified algorithms, task-shifting to nursing staff, and integration into existing newborn care pathways may further facilitate uptake and sustainability [83–87].

Together, these case studies from Rwanda, Nigeria, and other LMICs illustrate that while POS is not without challenges in resource-limited contexts, its low cost, ease of use, and dual capacity to detect both cardiac and non-cardiac conditions make it a valuable intervention. Adaptations tailored to local health system capacity such as simplified algorithms, task-shifting to nursing staff, and integration into existing newborn health programs may facilitate broader uptake and sustainability.

## Strategies to Improve Screening Impact

Congenital heart defects (CHD) remain one of the most significant contributors to neonatal morbidity and mortality worldwide, with nearly eight cases per 1000 live births. [88,89] Among these, the subset classified as Critical Congenital Heart Defects (CCHD) poses an especially urgent threat, as delayed detection can result in severe physiological compromise and mortality within days after birth. Pulse oximetry screening has emerged as a practical, non-invasive, and highly specific tool to facilitate early diagnosis, though its moderate sensitivity means some cases may still be missed. Studies have consistently shown the dangers of delayed diagnosis. In the U.S. National Birth Defects Prevention Study, nearly 30% of infants with CCHD were identified more than three days after birth, underscoring the life-saving potential of routine hospital-based screening programs [90]. Equally, analysis of reporting practices in California revealed substantial inconsistencies, with over one-third of hospitals not submitting screening data, potentially missing hundreds of CCHD cases annually. These findings emphasize that, beyond the adoption of screening technologies, systematic policy, standardized workflows, and data integrity are crucial for maximizing the impact of newborn screening [91].

The integration of electronic medical records (EMRs) and computerized clinical decision support (CCDS) systems into newborn screening programs offers promising solutions to bridge these gaps. A retrospective study from the United Arab Emirates demonstrated that embedding a fully automated EMR-driven protocol into the routine nursing workflow achieved a 98.9% compliance rate in CCHD screening, while significantly improving early disease identification. This echoes similar findings from other specialties: the implementation of an EMR-based COPD flowsheet in a United States tertiary setting enhanced compliance with evidence-based guidelines, improving the use of validated severity assessment tools, vaccination rates, and inhaler education. These experiences highlight the transformative role of EMR-driven systems in both acute and chronic disease management, reducing variability in care and promoting adherence to standardized guidelines. Furthermore, CCDS technology extends these benefits by providing real-time prompts and logical decision-making algorithms that can guide clinicians, mitigate missed diagnoses, and enhance care quality. When adapted to CCHD screening, such digital integration could ensure not only comprehensive coverage but also timely escalation of positive cases through predefined clinical pathways.

Importantly, EMR-supported screening solutions can be particularly powerful in resource-limited settings where infrastructure and follow-up systems are constrained. Evidence from Kenya illustrates the feasibility of training community health workers to conduct hearing screenings using a smartphone-based EMR and video-otoscopy platform. Despite limited medical backgrounds, these workers were able to generate reliable EMRs, identify pathology, and facilitate referral of affected children with no major disruptions to workflow. The success of this mobile-health strategy offers valuable lessons for CHD screening in low- and middle-income countries (LMICs): digital platforms can extend the reach of pulse oximetry, standardize documentation, and enable remote consultation when specialized expertise is unavailable locally. Taken together, the global evidence base shows that screening programs built upon robust policy mandates, EMR integration, and decision-support tools not only increase compliance and data reliability but also enhance diagnostic timeliness and overall neonatal outcomes. As healthcare systems worldwide continue to expand newborn screening, embedding technology into workflows represents a critical step toward achieving sustainable, high-quality, and equitable detection of life-threatening conditions such as CHD [92,93].

## Conclusions

Pulse oximetry screening (POS) has emerged as a valuable tool for the early detection of congenital heart defects (CCHD), complementing prenatal ultrasound and physical examination. Large-scale studies and meta-analyses have demonstrated that POS has high specificity and moderate sensitivity, with the ability to detect life-threatening cardiac malformations such as hypoplastic left heart syndrome, transposition of the great arteries, and pulmonary atresia before the onset of clinical decompensation. Importantly, evidence suggests that its implementation reduces diagnostic delays, prevents morbidity related to circulatory collapse, and improves neonatal outcomes through timely referral for definitive interventions.

Despite these benefits, several limitations remain. Pulse oximetry is not a standalone diagnostic tool and is susceptible to false negatives, particularly in lesions with balanced systemic and pulmonary circulation or delayed hypoxemia, such as coarctation of the aorta or interrupted aortic arch. False positives may also occur in cases of pulmonary disease, sepsis, or persistent pulmonary hypertension of the newborn. These diagnostic uncertainties underscore the need for POS to be integrated into a broader screening framework that includes prenatal detection, thorough clinical examination, and appropriate confirmatory echocardiography.

The implementation of POS varies considerably across clinical settings. In high-resource healthcare systems, screening is often standardized, supported by electronic health record integration, and performed by trained nursing staff with access to confirmatory echocardiography. In contrast, low- and middle-income countries (LMICs) face significant challenges. These include limited access to pulse oximeters, inadequate training of healthcare personnel, delayed referral pathways, and limited availability of pediatric cardiology services. Additionally, regional disparities in healthcare infrastructure may result in inconsistent adoption, particularly in rural or underserved areas. These barriers highlight the need for context-specific strategies that balance accuracy, feasibility, and sustainability.

From a public health perspective, POS represents one of the most cost-effective interventions for reducing mortality from undiagnosed CHD. The integration of POS into routine newborn screening has been endorsed by organizations such as the AHA and AAP in the United States, and many countries have followed suit with national guidelines. However, equitable implementation remains an ongoing concern. Populations in resource-limited settings are at greater risk of missed or delayed diagnoses due to systemic barriers, potentially exacerbating global disparities in CHD outcomes. Addressing these inequities requires coordinated efforts in health policy, capacity building, and international collaboration.

Looking forward, research and practice should prioritize refining screening protocols to maximize sensitivity while minimizing unnecessary referrals. Innovations such as dual-site screening (pre- and post-ductal measurements), algorithm-based interpretation, and integration with telemedicine platforms may enhance diagnostic accuracy and extend the reach of screening programs. Additionally, evaluating long-term outcomes—including cost-effectiveness, parental anxiety, and system-level impact—will be critical in guiding sustainable implementation strategies.

In conclusion, pulse oximetry screening represents a simple, non-invasive, and effective approach to the early detection of CHD. While its clinical utility is well established, challenges in implementation, particularly in resource-limited settings, continue to limit its global impact. Future progress will depend on overcoming logistical barriers, strengthening health system capacity, and ensuring equitable access to screening and follow-up care. By addressing these challenges, POS has the potential to become a truly universal standard of care, improving survival and quality of life for infants with congenital heart defects worldwide.

## Figures and Tables

**Figure 1:**
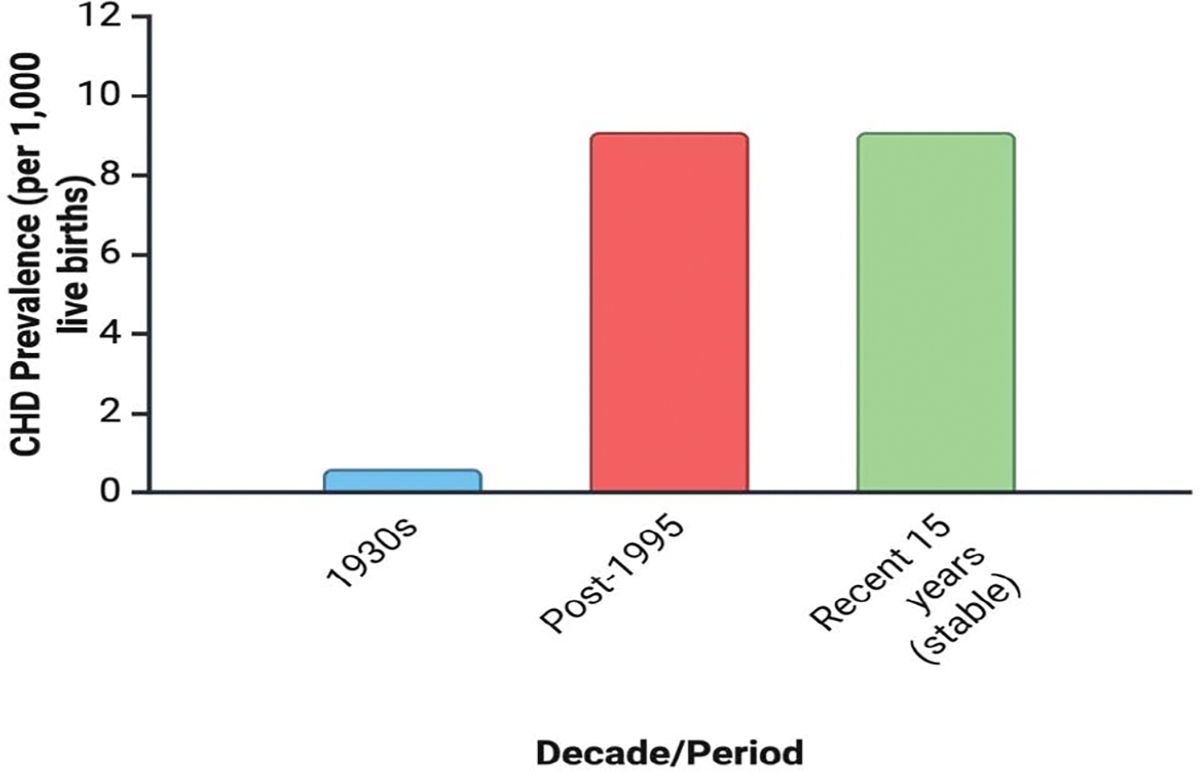
Temporal trends in the global birth prevalence of congenital heart defects (CHD). Birth prevalence of CHD increased substantially over the past century, rising from 0.6 per 1,000 live births (95% CI: 0.4–0.8) in the 1930s to 9.1 per 1,000 (95% CI: 9.0–9.2) after 1995, before stabilizing over the last 15 years at approximately 1.35 million affected newborns annually. The upward trend is primarily attributed to advances in diagnostic technology and screening programs, although genetic, environmental, and socio-economic factors may also contribute.

**Figure 2:**
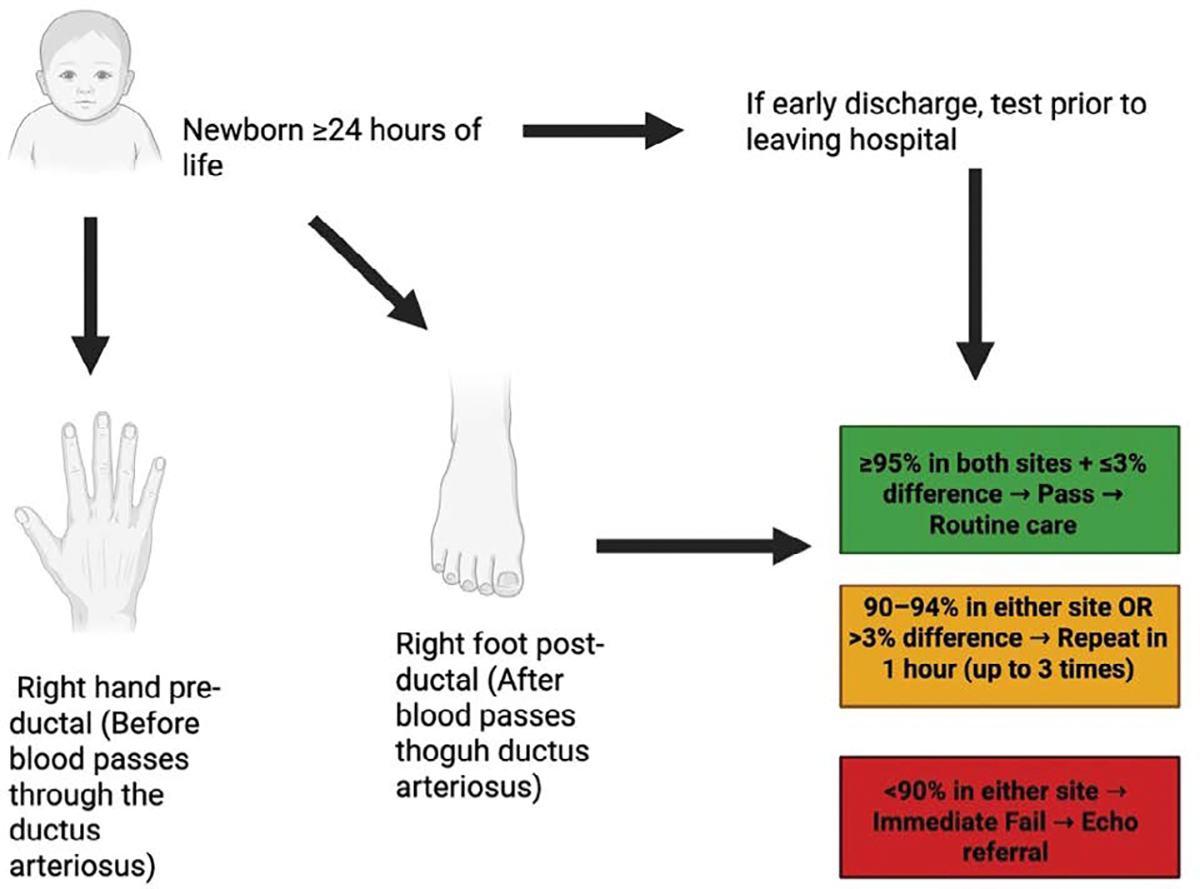
Algorithm for Pulse Oximetry Screening (POS) in Newborns. Screening is recommended after 24 hours of life to reduce false positives from transitional circulation, though testing prior to discharge may be performed if early discharge is anticipated. Oxygen saturation is measured at two sites: the right hand (pre-ductal) and either foot (post-ductal). A result is considered a pass if both sites are ≥95% and the pre-/post-ductal difference is ≤3%. Readings of 90–94% in either site or a >3% difference prompt repeat testing at 1-hour intervals for up to three attempts. An oxygen saturation <90% in either site is an immediate failure, warranting prompt referral for echocardiography to evaluate for critical congenital heart disease.

## Key Points, Challenges, and Outstanding Questions

- Pulse oximetry screening (POS) is a non-invasive, inexpensive, and highly specific tool that significantly improves early detection of congenital heart defects, complementing prenatal ultrasound and physical examination.
- Since its universal adoption in the United States in 2011, POS has reduced delayed diagnoses, emergency hospitalizations, and infant mortality from CHD.
- POS can also detect other hypoxemic conditions such as neonatal sepsis, pneumonia, and persistent pulmonary hypertension, broadening its clinical utility.
- Cost-effectiveness studies indicate that POS is economically sustainable in high- and middle-income countries, especially when integrated into existing newborn screening programs.
- Pilot studies in Rwanda, Nigeria, and India have demonstrated POS feasibility in low-resource settings, showing benefits beyond cardiac disease detection.
- Integration with electronic medical records and decision-support systems enhances compliance, reduces human error, and standardizes protocols.
- POS has limited sensitivity for certain lesions, particularly coarctation of the aorta and interrupted aortic arch, the places for later manifestation of hypoxemia.
- Protocol variability in timing (24–72 hours), measurement sites (pre- vs post-ductal), and repeat testing thresholds leads to inconsistent outcomes across studies.
- Evidence suggests racial bias in pulse oximetry accuracy, with overestimation of oxygen saturation in infants with darker skin tones, raising concerns about equity.
- Implementation barriers in low- and middle-income countries include limited echocardiography access, poor referral systems, shortages of trained personnel, and high rates of home births.
- Fragmented state- and hospital-level reporting (e.g., in the United States) limits national surveillance, outcome assessment, and quality improvement.
- How can POS protocols be adapted to minimize disparities across racial groups, rural versus urban populations, and high- versus low-resource healthcare systems?
- What protocol optimizations (e.g., dual-site vs single-site testing, repeat testing frequency) provide the best balance between sensitivity and specificity?
- How can POS be integrated most effectively with prenatal diagnosis, physical exam, telemedicine, and digital health tools to form a multilayered screening framework?
- What scalable models (district/state scale-up, task-shifting, public–private partnerships) are most feasible for sustainable expansion in low- and middle-income countries?
- Beyond immediate survival, what are the long-term neurodevelopmental, healthcare cost, and family outcomes associated with early CHD detection through POS?
- Should POS be formalized as a universal newborn screening standard globally, and how can international policy collaboration support equitable implementation?
